# DRP1 inhibition rescues retinal ganglion cells and their axons by preserving mitochondrial integrity in a mouse model of glaucoma

**DOI:** 10.1038/cddis.2015.180

**Published:** 2015-08-06

**Authors:** K-Y Kim, G A Perkins, M S Shim, E Bushong, N Alcasid, S Ju, M H Ellisman, R N Weinreb, W-K Ju

**Affiliations:** 1 Department of Neuroscience, Center for Research in Biological Systems, National Center for Microscopy and Imaging Research, University of California, San Diego, La Jolla, CA, USA; 2 Laboratory for Optic Nerve Biology, Department of Ophthalmology, Hamilton Glaucoma Center, University of California, San Diego, La Jolla, CA, USA

## Abstract

Glaucoma is the leading cause of irreversible blindness and is characterized by slow and progressive degeneration of the optic nerve head axons and retinal ganglion cell (RGC), leading to loss of visual function. Although oxidative stress and/or alteration of mitochondrial (mt) dynamics induced by elevated intraocular pressure (IOP) are associated with this neurodegenerative disease, the mechanisms that regulate mt dysfunction-mediated glaucomatous neurodegeneration are poorly understood. Using a mouse model of glaucoma, DBA/2J (D2), which spontaneously develops elevated IOP, as well as an *in vitro* RGC culture system, we show here that oxidative stress, as evidenced by increasing superoxide dismutase 2 (SOD2) and mt transcription factor A (Tfam) protein expression, triggers mt fission and loss by increasing dynamin-related protein 1 (DRP1) in the retina of glaucomatous D2 mice as well as in cultured RGCs exposed to elevated hydrostatic pressure *in vitro*. DRP1 inhibition by overexpressing DRP1 K38A mutant blocks mt fission and triggers a subsequent reduction of oxidative stress, as evidenced by decreasing SOD2 and Tfam protein expression. DRP1 inhibition promotes RGC survival by increasing phosphorylation of Bad at serine 112 in the retina and preserves RGC axons by maintaining mt integrity in the glial lamina of glaucomatous D2 mice. These findings demonstrate an important vicious cycle involved in glaucomatous neurodegeneration that starts with elevated IOP producing oxidative stress; the oxidative stress then leads to mt fission and a specific form of mt dysfunction that generates further oxidative stress, thus perpetuating the cycle. Our findings suggest that DRP1 is a potential therapeutic target for ameliorating oxidative stress-mediated mt fission and dysfunction in RGC and its axons during glaucomatous neurodegeneration. Thus, DRP1 inhibition may provide a new therapeutic strategy for protecting both RGCs and their axons in glaucoma and other optic neuropathies.

Excessive mitochondrial (mt) fission-mediated dysfunction has been implicated in various neurodegenerative diseases.^1, 2, 3, 4, 5^ Complexes of dynamin-related protein 1 (DRP1) assemble from the cytosol onto the mitochondria at focal sites of mt fission and regulate mt fission.^6, 7^ Recent evidence indicates that posttranslational modifications of DRP1 are linked to mt dysfunction-mediated bioenergetic failure, synaptic injury and neuronal cell death.^2, 3, 8, 9, 10^ Phosphorylation of DRP1 at serine 616 (S616) by cyclin-dependent kinase 1/cyclin B triggers increased activity of mt fission.^11, 12^ However, phosphorylation of DRP1 at serine 637 by the cyclic AMP-dependent protein kinase inhibits mt fission by decreasing DRP1 activity.^13, 14, 15^ Although it is not well understood whether DRP1 and DRP1 S616 phosphorylation have critical roles in mt fission-mediated neurodegeneration of the central nervous system (CNS), inhibition of DRP1 activity prevents mt fission and protects against neuronal cell death.^3, 16, 17, 18, 19^

Glaucoma is the leading cause of irreversible blindness and affects 70 million people worldwide.^20, 21^ Intraocular pressure (IOP) is a major, and perhaps the most significant, risk factor for loss of visual function from glaucoma. Moreover, IOP is the only modifiable risk factor and reduction of IOP is the standard treatment. However, lowering IOP itself is not always effective for preserving visual function in patients with primary open-angle glaucoma (POAG).^20, 21^ Recent studies have demonstrated that oxidative stress-mediated mt dysfunction is associated with glaucomatous neurodegeneration.^22, 23, 24, 25, 26^ Furthermore, growing evidence indicates that impairment of mt dynamics may contribute to the pathogenesis in experimental rodent models of glaucoma^1, 27, 28^ as well as in patients with POAG.^29, 30, 31, 32^ Regardless, the mechanisms of oxidative stress-mediated consequences of impaired mt dynamics and function, and subsequent retinal ganglion cell (RGC) soma and axon degeneration in glaucomatous neurodegeneration, remain unknown.

We test whether oxidative stress induced by IOP elevation triggers DRP1 and DRP1 S616 phosphorylation-mediated mt fission in the RGC and probe for degeneration in its axon using the glaucomatous DBA/2J (D2) mouse that spontaneously develops elevated IOP.^1, 33, 34^ Furthermore, we investigate whether DRP1 inhibition by overexpressing the dominant-negative DRP1 K38A mutant (DRP1^K38A^) promotes RGC survival and axon preservation by maintaining mt integrity. Here we show that oxidative stress triggers mt fission and loss by increasing DRP1 activity in glaucomatous RGCs. Furthermore, DRP1 inhibition blocks oxidative stress and rescues RGCs and their axons by preserving mt integrity, suggesting that DRP1 could be a potential therapeutic target for ameliorating oxidative stress-mediated mt fission and dysfunction in glaucomatous RGC degeneration.

## Results

### Oxidative stress increases total protein expression of DRP1 but not its S616 phosphorylation in the glaucomatous retina

**Figure 1:**
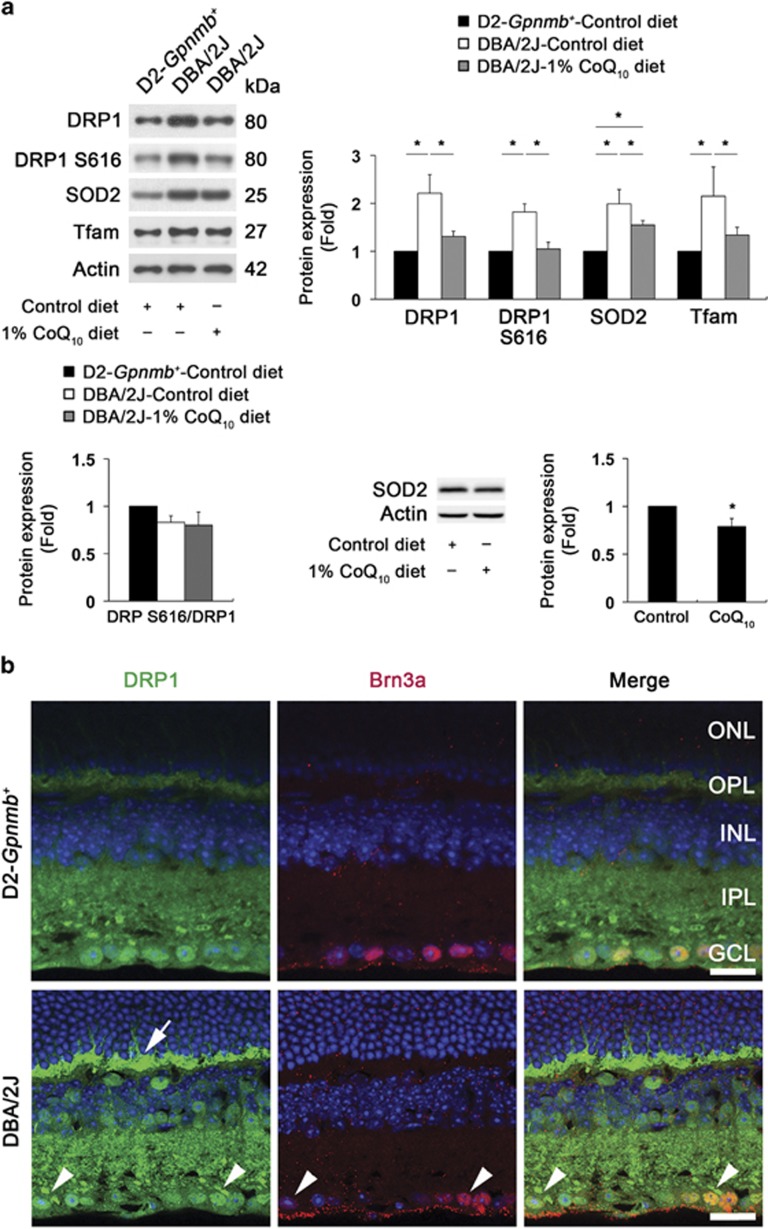

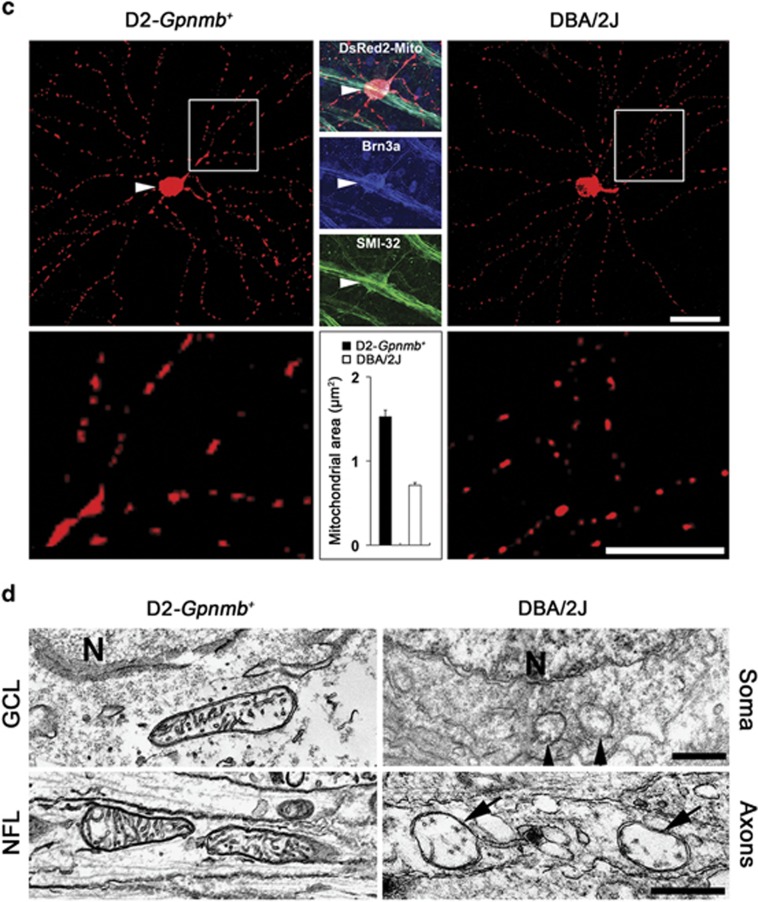
Oxidative stress increases DRP1 but not its phosphorylation at Ser616, and triggers mt fission in RGC somas and their axons of glaucomatous D2 mice. (**a**) The retinas of glaucomatous D2 mice had significantly increased total DRP1 protein expression, as well as SOD2 and Tfam protein expression. However, the CoQ_10_ diet significantly decreased total DRP1 protein expression, as well as SOD2 and Tfam protein expression in the retinas of glaucomatous D2 mice. Interestingly, DRP1 S616 phosphorylation was not changed among experimental groups. In addition, the CoQ_10_ diet significantly decreased SOD2 protein expression in the retina of D2-*Gpnmb*^*+*^ mice compared with D2-*Gpnmb*^*+*^ mice treated with control diet. Values are mean±S.D. *Significant at *P*<0.05 compared with D2-*Gpnmb*^*+*^mice treated with control diet or glaucomatous D2 mice treated with control diet. (**b**) The retina of glaucomatous D2 mouse had increased DRP1 immunoreactivity in the OPL (arrow), as well as INL and GCL compared with D2-*Gpnmb*^*+*^mouse. It is noteworthy that neurons (arrows) containing accumulated DRP1 immunoreactivity in the cytosolic and perinuclear region co-labeled by Brn3a immunoreactivity in the GCL of the glaucomatous retina. Scale bar: 20 *μ*m (all panels). (**c**) Mitochondrial morphology was assessed in the retina by transduction of AAV2-pDsRed2-Mito. Using double immunohistochemistry for Brn3a and SMI-32, DsRed2-Mito is expressed in the mitochondria of RGC somas that were positive for Brn3a, as well as in the mitochondria of its axon and dendrites that were positive for SMI-32 in D2-*Gpnmb*^*+*^ mice. Glaucomatous D2 mice contained relatively shorter mitochondria in the RGC axon and dendrites compared with the D2-*Gpnmb*^*+*^ mouse. Note that mt area analysis showed about 53% mt area loss. Scale bar: 20 *μ*m (all panels). (**d**) In comparison with D2-*Gpnmb*^*+*^mouse, electron microscopy of glaucomatous D2 mice showed small rounded mitochondria also showing cristae depletion (arrows) in the RGC soma in the GCL and its axon in the NFL. OPL, outer plexiform layer; INL, inner nuclear layer; IPL, inner plexiform layer; GCL, ganglion cell layer; NFL, nerve fiber layer. Scale bar: 1 *μ*m (all panels)

To determine whether oxidative stress induced by elevated IOP alters the expression levels of DRP1 and its S616 phosphorylation in the glaucomatous retina, D2-*Gpnmb*^*+*^ and pre-glaucomatous D2 mice were fed with coenzyme Q10 (CoQ_10_, 1%), a potent antioxidant,^23, 26, 35^ or a control diet daily for 5 months and we examined the protein expression levels of superoxide dismutase 2 (SOD2) and mt transcription factor A (Tfam), as well as DRP1 and DRP1 S616 phosphorylation. We found that the CoQ_10_ diet significantly decreased total DRP1 as well as SOD2 and Tfam protein expression in the glaucomatous retina (*P*<0.05; Figure 1a). Of interest, however, there were no changes of the expression level of DRP1 S616 phosphorylation normalized by total DRP1 (Figure 1a). In addition, we found that the CoQ_10_ diet significantly decreased SOD2 protein expression by 0.79±0.08-fold in the retina of D2-*Gpnmb*^*+*^ mice (*P*<0.05; Figure 1a); this confirms that the level of oxidative stress is decreased following CoQ_10_ treatment. We also found that DRP1 immunoreactivity was increased in the outer plexiform layer (OPL) and inner retinal layers of the glaucomatous retina (Figure 1b), accompanied by elevated IOP-induced RGC loss (*P*<0.05; Supplementary Figures S1a and b, and Supplementary Table S1). Of note, neurons containing accumulated DRP1 immunoreactivity in the cytosolic and perinuclear regions were co-labeled with Brn3a-positive RGCs in the ganglion cell layer (GCL) of the glaucomatous retina (Figure 1b).

To further determine whether elevated pressure specifically induces oxidative stress as well as increases the expression levels of DRP1 and DRP1 S616 phosphorylation in RGCs, we next probed for these changes using cultured RGCs exposed to elevated hydrostatic pressure *in vitro*. Consistent with our *in vivo* results, we found that elevated pressure significantly increased reactive oxygen species (ROS) generation and SOD2 protein expression in cultured RGCs, accompanied by decreased cell viability (*P*<0.05; Supplementary Figures S2a–g and Supplementary Table 2). Of interest, elevated pressure significantly increased the total protein expression of DRP1 but not its S616 phosphorylation in cultured RGCs (Supplementary Figure S3a). In parallel, these results were supported by increased DRP1 immunoreactivity in Brn3a-positive pressurized RGCs (Supplementary Figure S3b).

### Mitochondrial fission, loss and mitophagosome formation occurs in glaucomatous RGCs and their axons

To examine mt fission in the RGC somas and their axons *in vivo*, we injected a recombinant adeno-associated virus serotype 2 (AAV2) carrying pDsRed2-Mito into the eyes of D2-*Gpnmb*^*+*^ and pre-glaucomatous D2 mice. In comparison with the D2-*Gpnmb*^*+*^ mouse, we found that the glaucomatous D2 mouse contained relatively shorter mitochondria in the RGC axon and dendrites (Figure 1c), showing about 53% mt area loss (Figure 1c, graph in the middle of lower panel). In support of these findings, transmission electron microscopy analysis showed that the RGC soma in the GCL and its axon in the nerve fiber layer of glaucomatous D2 mouse contained small fragmented mitochondria (Figure 1d), whereas the D2-*Gpnmb*^*+*^ mouse contained a tubular form of elongated mitochondria in both layers (Figure 1d).

**Figure 2:**
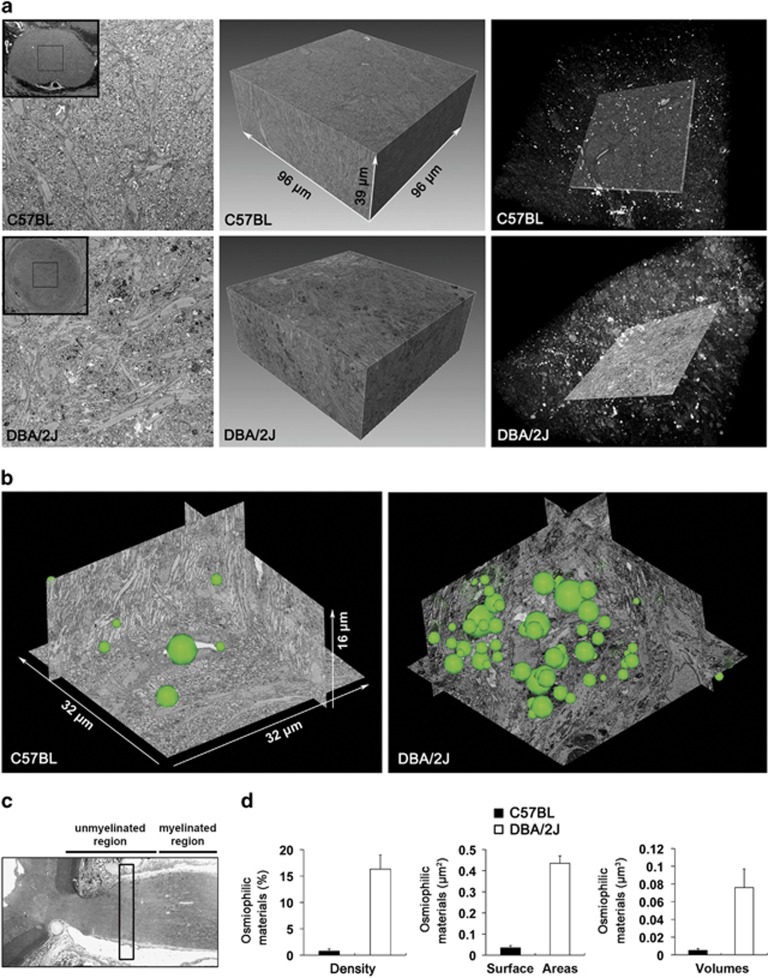
3D reconstruction of evulsions in the axons of glial lamina in glaucomatous D2 mice. (**a**) Representative SBEM single sections (left panels) from SBEM volumes (middle panels) containing a total of 200 slices at 80-nm section thickness in the glial lamina of C57BL and glaucomatous D2 mice show striking differences in the occurrence of evulsions, protrusions and lipid droplets. The MIP of SBEM volumes are displayed with inverted image contrast (white is highest EM signal, black is no signal, right panels). Note the greater content of white osmiophilic material in the D2 sample. (**b**) Representative reconstructions of evulsions (green spheres) in the glial lamina of C57BL and glaucomatous D2 mice. (**c**) Schematic representation with the glial lamina region of immunohistochemical data collection. (**d**) Quantitative analysis of density, surface areas and volumes of osmiophilic materials in the glial lamina of glaucomatous D2 mice. Values are mean±S.E.M.

To further determine the relationship between axonal degeneration and mt fission in the glial lamina of glaucomatous D2 mice, we first assessed axonal degeneration by measuring the maximum intensity projection (MIP) of serial block-face scanning electron microscopy (SBEM) volume rendering and measured density, surface areas and volumes. In comparison with the C57BL mouse (Figures 2a–d and Supplementary Movie S1), representative images from SBEM volumes from the glaucomatous glial lamina contained a higher number of dense deposits in degenerative axons as well as hypertrophic astroglial activation (Figures 2a and b, and Supplementary Movie S2). Interestingly, the MIP of SBEM volume rendering from the glaucomatous glial lamina contained randomly osmiophilic lipid materials including evulsions, protrusions and lipid droplets (Figures 2a and b, and Supplementary Movie S2). Moreover, the axons in the glaucomatous glial lamina contained higher levels of osmiophilic lipid materials through increased density, surface areas and volumes (Figures 2b–d).

**Figure 3:**
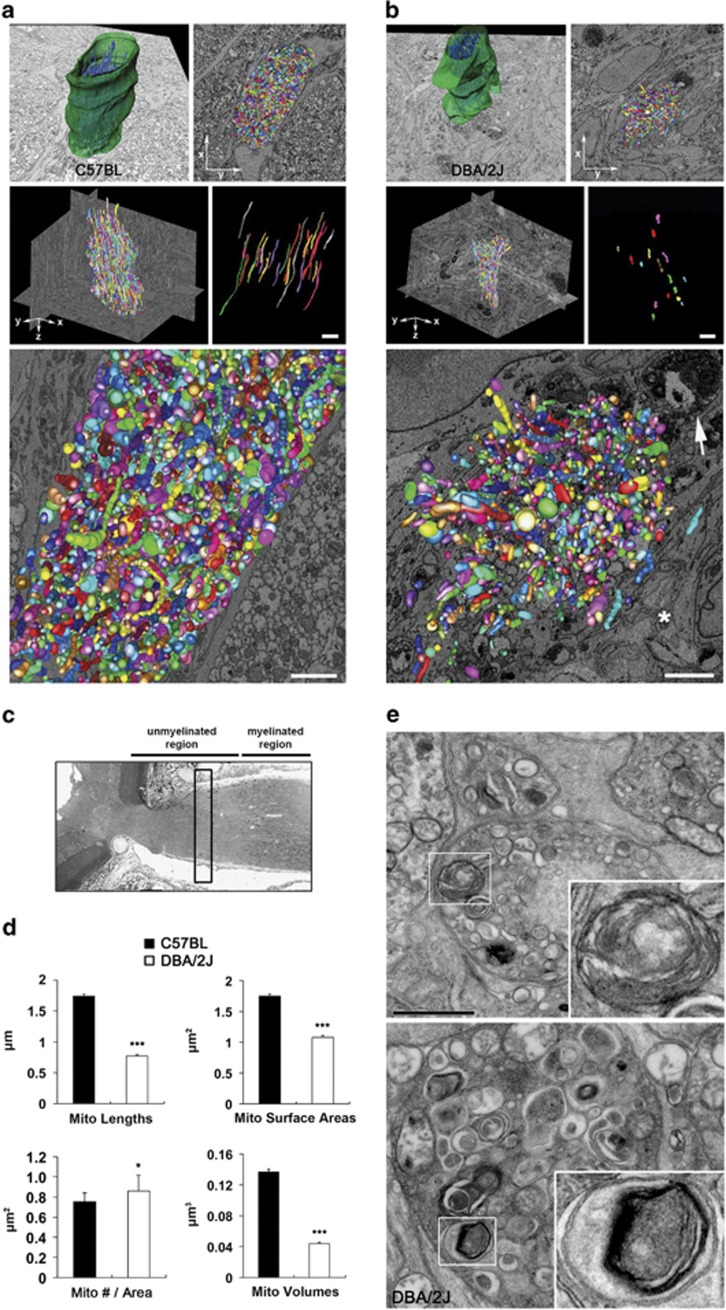
3D reconstruction of mt fission in the axons of glial lamina in glaucomatous D2 mice. (**a** and **b**) Mitochondrial segmentation from SBEM volumes. Reconstructed mitochondria (blue) in each axon bundle (green) of the glial lamina from C57BL and glaucomatous D2 mice. SBEM stacks showed 1951 (**a**) and 438 (**b**) traced mitochondria emerging as randomly variously colored objects in different dimension views in the glial lamina from C57BL and glaucomatous D2 mice, respectively. Representative mitochondria are in various colors and glaucomatous D2 mouse shows mt fission and loss, as well as an evulsion formation (arrow) in the axons and hypertrophic astroglial activation (asterisk) in the glial lamina. Right: examples of small subsets of adjacent mitochondria highlighting the differences in lengths. Scale bar: 1 *μ*m (middle); 2 *μ*m (all panels in the bottom). (**c**) Schematic representation with the glial lamina region of SBEM data collection. (**d**) Quantitative analysis of mt lengths, surface areas, number per area and volumes in the axons of the glial lamina. Values are mean±S.E.M. *Significant at *P*<0.05 and ***significant at *P<*0.001 compared with C57BL mice. (**e**) Autophagy of mitochondria (mitophagy) is evident in the axons of the glial lamina of glaucomatous D2 mice. The left panel shows an EM image of an evulsion containing degrading vacuoles in the axons of the glial lamina and the right panel shows an EM image of an even larger evulsion showing an increased number of degrading vacuoles. Both insets show a mitophagosome engulfing a degraded mitochondrion. Scale bar: 2 *μ*m (all panels)

We next assessed mt fission by measuring mt lengths, surface areas and volume density, as well as mitophagosome formation in the axons of the glial lamina in glaucomatous D2 mice using a three-dimensional (3D) reconstruction of the axon bundle and mitochondria produced by SBEM data sets. In comparison with the C57BL mouse (Figure 3a and Supplementary Movie S3), representative images of mt segmentation from SBEM stacks showed excessive mt fission and loss in the axons, accompanied by a representative evulsion formation and hypertrophic astroglial activation, of the glaucomatous glial lamina (Figure 3b and Supplementary Movie S4). In addition, quantitative analysis showed significant decreases of mt lengths (0.77±0.02 *μ*m), surface areas (1.08±0.03 *μ*m^2^) and volume density (0.04±0.001 *μ*m^3^) in the axons of the glial lamina in glaucomatous D2 mouse compared with the C57BL mouse (1.75±0.04 *μ*m, mt lengths; 1.74±0.04 *μ*m^2^, surface areas; and 0.14±0.003 *μ*m^3^, volume density), yet an increase in mt number (0.86±0.16 *μ*m^2^ *versus* 0.75±0.09 *μ*m^2^) (*P*<0.001; Figures 3c and d). Moreover, we found evidence of evulsions showing an increased number of degrading vacuoles and mitophagosomes engulfing degraded mitochondria in the axons of the glaucomatous glial lamina (Figures 3b and e), collectively suggesting that there are excessive mt fission-mediated loss in glaucomatous RGC axon degeneration.

Consistent with our *in vivo* results, we found mt fission in pressurized RGC somas *in vitro* (Supplementary Figures S4a–c). Quantitative analysis showed that the number of mitochondria was significantly increased to 1.5±0.57 in pressurized RGCs (*n*=20) compared with non-pressurized control (0.86±0.3, *n*=20) (*P*<0.001; Supplementary Figure 4b). In contrast, mt lengths were significantly decreased to 407±244 nm in pressurized RGCs (*n*=174) compared with non-pressurized control (765±492 nm, *n*=182) (*P*<0.05; Supplementary Figure S4b). However, there was no difference in mt volume density in cultured RGCs (Supplementary Figure S4b). The 3D tomographic reconstructions showed detailed mt membrane structure including the packing arrangement, shape and density of the cristae (Supplementary Figure S4c). Most of the cristae in control cells have both tubular and lamellar compartments. However, some cristae are completely lamellar or completely tubular. In the example shown, both lamellar and tubular cristae extended transversely. In contrast to the control mitochondrion (Supplementary Figure S4c and Supplementary Movie S5), a few lamellar cristae are also arranged longitudinally in a pressurized mitochondria (Supplementary Figure S4c and Supplementary Movie S6).

**Figure 4:**
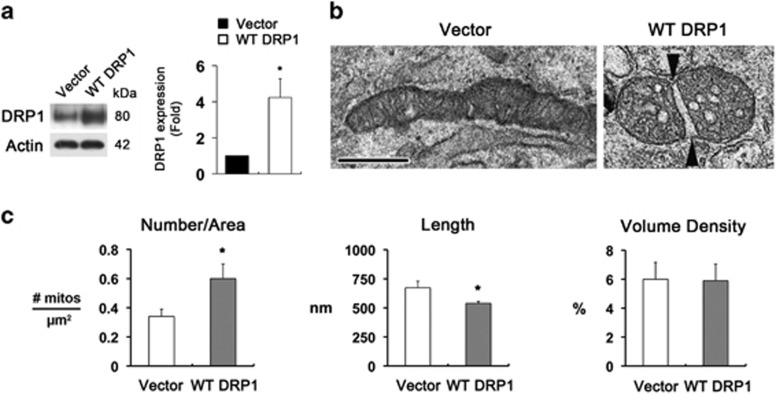
Increasing DRP1 triggers mt fission in RGCs *in vitro*. (**a**) Overexpression of WT DRP1 significantly increased DRP1 protein expression in cultured RGCs. Values are mean±S.D. *Significant at *P*<0.05 compared with control RGCs transfected with control vector. (**b**) Representative 2D images from transmission electron microscopy (TEM) analysis showed that the soma in cultured RGCs transfected with WT DRP1 had small rounded mitochondria and swollen cristae structure. Arrowheads point to opposite ends of a fission site. Scale bar: 500 nm (all panels). (**c**) Quantitative analysis showed that RGCs-transfected WT DRP1 showed increased number and decreased lengths of mitochondria. However, there was no difference in mt volume density between WT DRP1 and control. Values are mean±S.E.M. *Significant at *P*<0.05 compared with control RGCs transfected with control vector

To further confirm whether increasing DPR1 protein expression triggers mt fission in RGCs, we transfected the wild-type (WT) *DRP1* gene in cultured RGCs *in vitro*. We found that increasing DRP1 protein expression triggered mt fission with swollen cristae structure in the mitochondria of cultured RGCs (*P*<0.05; Figures 4a and b). Quantitative analysis showed that the number of mitochondria was significantly increased to 0.6±0.1 *μ*m^2^ in cultured RGCs transfected with WT DRP1 (*P*<0.001; Figure 4c). In contrast, mt lengths were significantly decreased to 539±16.8 nm in cultured RGCs transfected with WT DRP1 (*P*<0.05; Figure 4c). However, there was no difference in mt volume density between the two groups (Figure 4c), suggesting that DRP1 is a key factor of mt fission in RGCs.

### DRP1 inhibition protects RGCs by blocking oxidative stress and the apoptotic pathway

**Figure 5:**
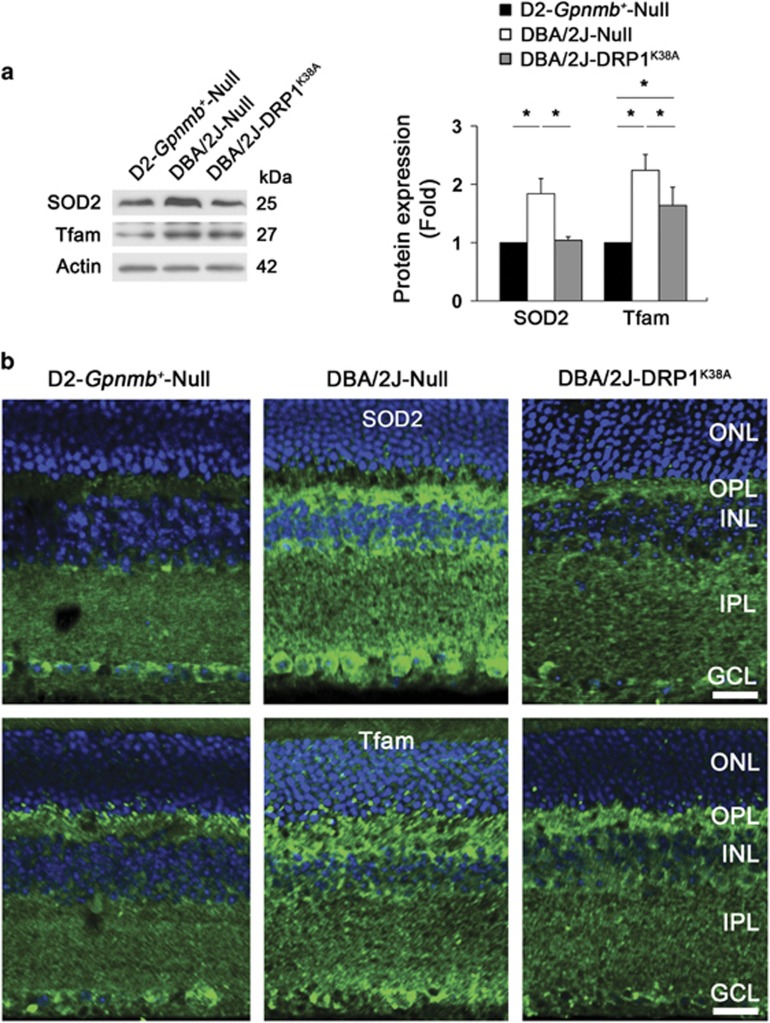
DRP1 inhibition protects RGCs against oxidative stress in glaucomatous D2 mice. (**a**) Overexpression of DRP1^K38A^ significantly decreased the levels of SOD2 and Tfam protein expression in the retinas of glaucomatous D2 mice. Values are mean±S.D. *Significant at *P*<0.05 compared with D2-*Gpnmb*^*+*^ transduced with AAV2-Null or glaucomatous D2 mice transduced with AAV2-Null. (**b**) Overexpression of DRP1^K38A^ decreased SOD2 and Tfam immunoreactivities in the OPL, INL, IPL and GCL. OPL, outer plexiform layer; INL, inner nuclear layer; IPL, inner plexiform layer; GCL, ganglion cell layer. Scale bar: 20 *μ*m (all panels)

To determine whether DRP1 inhibition blocks oxidative stress and apoptotic cell death in the glaucomatous retina, we injected AAV2 carrying a green fluorescent protein (GFP), Null or pDRP1^K38A^ into the eyes of D2-*Gpnmb*^*+*^ and pre-glaucomatous D2 mice (Supplementary Figures S5a and b). Using AAV2-GFP transduction and Brn3a whole-mount immunohistochemistry (Supplementary Figure S5c), we first found that the transduction efficiency of AAV2-GFP was 19.5±7.9% among the Brn3a-positive RGCs in glaucomatous D2 mice (Supplementary Figures S5d–g). With no significant difference in IOPs (Supplementary Figure S6), we found that DRP1 inhibition significantly decreased the levels of SOD2 and Tfam protein expression in the glaucomatous retina (*P*<0.05; Figure 5a). In addition, immunohistochemical analysis confirmed that DRP1 inhibition decreased SOD2 and Tfam immunoreactivities in the OPL and in neurons of the inner retinal layers of glaucomatous D2 mice (Figure 5b), suggesting the possibility that DRP1 inhibition blocks oxidative stress.

**Figure 6:**
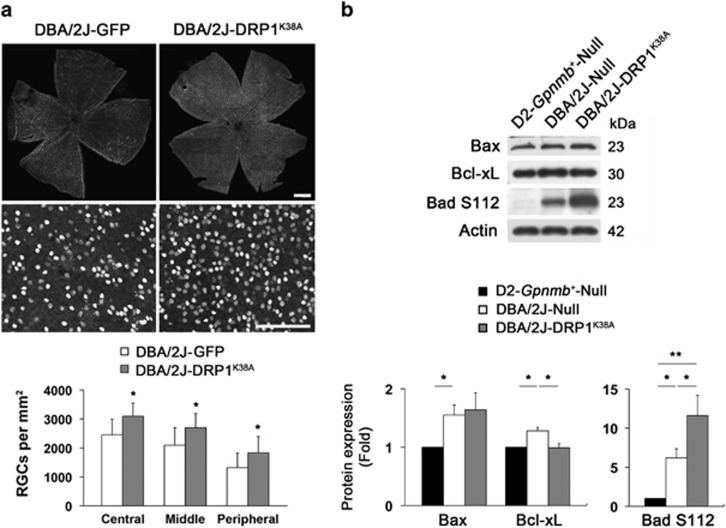
DRP1 inhibition protects RGCs by modulating the apoptotic pathway in glaucomatous D2 mice. (**a**) Quantitative analysis by whole-mount immunohistochemistry for Brn3a showed that overexpression of DRP1^K38A^ promoted RGC survival in glaucomatous D2 mice. Values are mean±S.D. *Significant at *P*<0.05 compared with D2-*Gpnmb*^*+*^ transduced with AAV2-GFP. Scale bar: 50 *μ*m (**a**, upper panels); 100 *μ*m (**a**, lower panels). (**b**) Overexpression of DRP1^K38A^ did not block increased Bax protein expression but significantly decreased the levels of Bcl-xL protein and Bad S112 phosphorylation in the retinas of glaucomatous D2 mice. Values are mean±S.D. *Significant at *P*<0.05 compared with D2-*Gpnmb*^*+*^ mice transduced with AAV2-Null or AAV2-GFP, or glaucomatous D2 mice transduced with AAV2-Null; **Significant at *P*<0.01 compared with D2-*Gpnmb*^*+*^ mice transduced with AAV2-Null

To further investigate whether DRP1 inhibition promotes RGC survival by modulating the apoptotic pathway, we examined the expression levels of Bax and Bcl-xL, as well as Bad phosphorylation at serine 112 (S112) in the retina of glaucomatous D2 mice. We found that DRP1 inhibition significantly promoted RGC survival by about 31% in glaucomatous retina (Figure 6a and Supplementary Table S3). In comparison with the D2-*Gpnmb*^*+*^mice, Bax protein expression was significantly increased in the glaucomatous retina transduced with AAV2-Null (*P*<0.05; Figure 6b). However, there was no difference between glaucomatous retinas transduced with AAV2-Null and AAV2-DRP1^K38A^ (Figure 6b). The levels of Bcl-xL and Bad S112 phosphorylation were significantly increased in the glaucomatous retina transduced with AAV2-Null (*P*<0.05; Figure 6b). Interestingly, DRP1 inhibition significantly restored Bcl-xL protein expression but showed greater increase of Bad S112 phosphorylation in the glaucomatous retina (*P*<0.05; Figure 6b).

### DRP1 inhibition rescues RGC axons by preserving mt integrity in glaucomatous glial lamina

**Figure 7:**
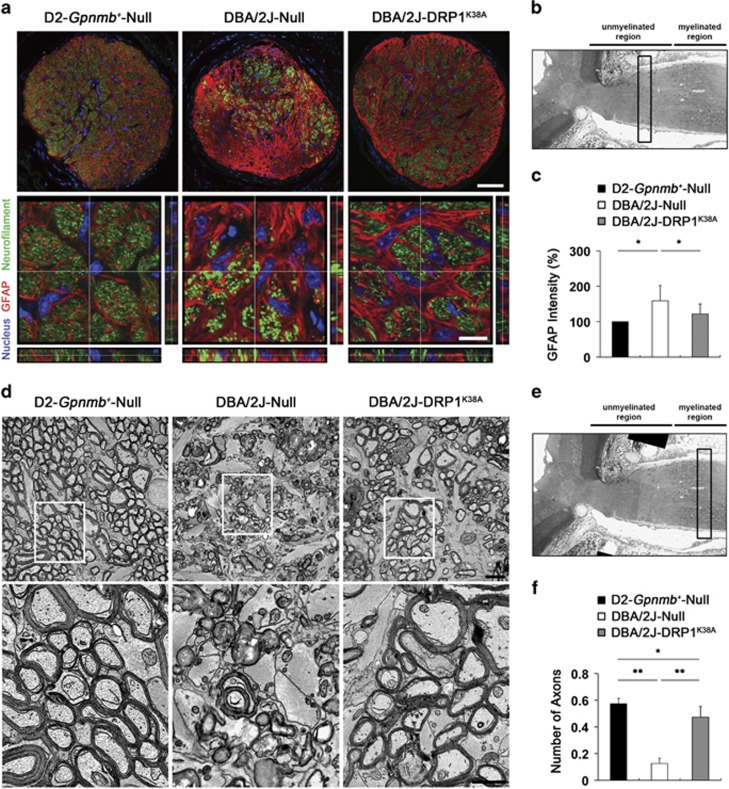
DRP1 inhibition prevents axonal degeneration and astroglial activation in the glial lamina of glaucomatous D2 mice. (**a**) GFAP and neurofilament double immunohistochemistry. Overexpression of DRP1^K38A^ partially preserved neurofilament and GFAP immunoreactivities in the glial lamina of glaucomatous D2 mice. Scale bar: 20 *μ*m (**a**, upper panels); 5 *μ*m (**a**, lower panels). (**b**) The origin of the cross-sections in **a** is shown in the glial lamina from the longitudinal section of D2-*Gpnmb*^*+*^ mice (open bar). (**c**) Quantitative analysis showed that overexpression of DRP1^K38A^ significantly preserved GFAP immunoreactivity in the glial lamina of glaucomatous D2 mice. Values are mean±S.D. *Significant at *P*<0.05 compared with D2-*Gpnmb*^*+*^ mice transduced with AAV2-Null or glaucomatous D2 mice transduced with AAV2-Null. Conventional transmission electron microscopy (TEM) analysis. (**d**) D2-*Gpnmb*^*+*^mice transduced with AAV2-Null showed normal healthy morphology of myelinated axons in the ON. In contrast, glaucomatous D2 mice transduced with AAV2-Null showed the absence of axons, as well as accumulation and disorganization of myelination in the ON. It is noteworthy that abundant hypertrophic astrocyte processes filled in the area of axon loss. However, overexpression of DRP1^K38A^ showed the preservation of axon structure and myelination in the ON of glaucomatous D2 mice. Scale bar: 2 *μ*m (all panels). (**e**) Schematic representation with the ON region of 2D EM data collection from the longitudinal section of D2-*Gpnmb*^*+*^ mice (bar). (**f**) Quantitative analyses showed that overexpression of DRP1^K38A^ significantly increased the number of axons in the ON of glaucomatous D2 mice (*n*=26 images). Values are mean±S.D. *Significant at *P*<0.05 compared with D2-*Gpnmb*^*+*^mice transduced with AAV2-Null; **Significant at *P*<0.01 compared with D2-*Gpnmb*^*+*^mice transduced with AAV2-Null or glaucomatous D2 mice transduced with AAV2-Null

Based on our observation of GFP expression in the axons of the glial lamina in pre-glaucomatous D2 mice (Supplementary Figures 7a and b), we next explored whether DRP1 inhibition rescues RGC axons by blocking mt fission in the glial lamina of glaucomatous D2 mice. In comparison with the D2-*Gpnmb*^*+*^ mice (Figures 7a and b), we first found greater loss of RGC axons by decreasing neurofilament immunoreactivity as well as hypertrophic astroglial reaction by increasing glial fibrillary acidic protein (GFAP) immunoreactivity in the glaucomatous glial lamina transduced with AAV2-Null (Figures 7a and c). However, of interest, DRP1 inhibition partially restored neurofilament immunoreactivity and blocked astrocytic activation in the glaucomatous glial lamina (Figures 7a and c).

To further confirm whether DRP1 inhibition preserves axon integrity, we next counted the number of axons in the optic nerve (ON) after the myelination transition zone (MTZ) in glaucomatous D2 mice. In comparison with the D2-*Gpnmb*^*+*^ mice, the glaucomatous D2 mouse transduced with AAV2-Null showed the absence of axons as well as accumulation and disorganization of myelination in the ON. In addition, abundant hypertrophic astrocyte processes filled in the area of axon loss (Figures 7d and e). Quantitative analysis showed that DRP1 inhibition significantly increased the number of axons in the ON, accompanied by preserving axons and their myelination in glaucomatous D2 mice (0.47±0.08/mm^2^; *P*<0.01) compared with glaucomatous D2 mice transduced with AAV2-Null (0.13±0.04/mm^2^) (Figure 7f).

**Figure 8:**
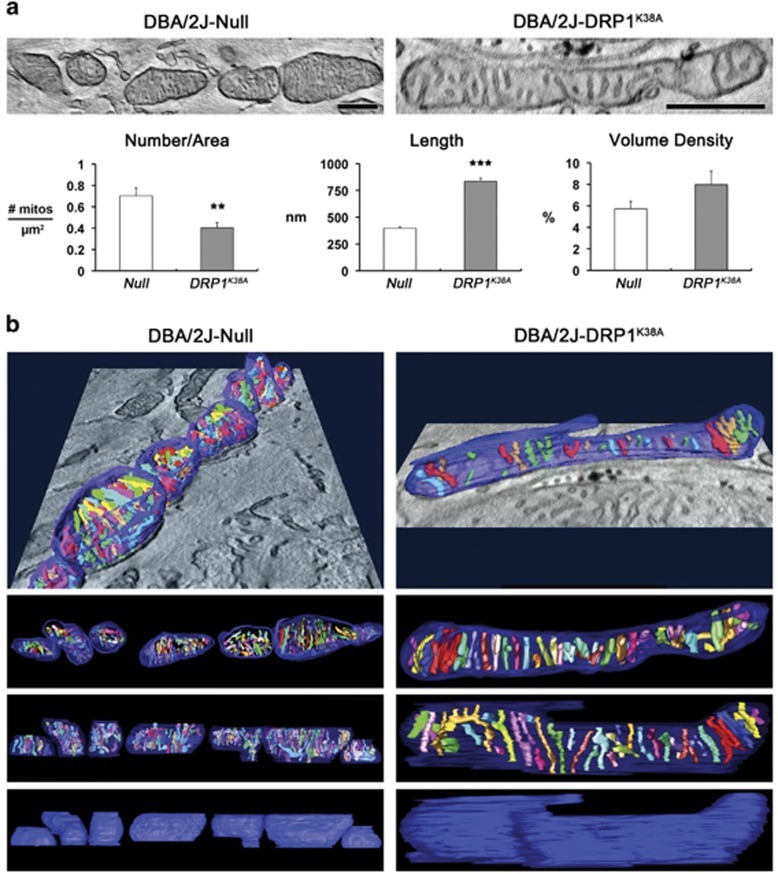
DRP1 inhibition preserves mt morphology in the axons of glial lamina in glaucomatous D2 mice. Electron tomography generated high-resolution 3D reconstructions of mitochondria in the glial lamina of glaucomatous D2 mice transduced with AAV2-Null or AAV2-DRP1^K38A^. (**a**) Slices (1.36-nm thick) through the middle of EM tomographic volumes of mitochondria are shown. Quantitative analysis of overexpressed DRP1^K38A^ showed increase of mt number but decrease of mt lengths in RGC axons of the glial lamina in glaucomatous D2 mice. However, there was no statistical difference in mt volume density. Values are mean±S.E.M. **Significant at *P*<0.01 and ***Significant at *P*<0.001 compared with glaucomatous D2 mice transduced with AAV2-Null. Scale bar: 500 nm (all panels). (**b**) Surface-rendered volumes of the segmented mitochondria provide information concerning shape and cristae architecture. The outer mt membrane is shown in blue (made translucent to better visualize the cristae) and cristae are in various colors

On the basis of these findings of DRP1 inhibition-mediated restoration of axonal integrity and glial reaction in glaucomatous glial lamina, we further determined whether DRP1 inhibition preserves the structural integrity of mitochondria in the axons of the glia lamina in glaucomatous D2 mice. Surprisingly, we found that DRP1 inhibition restored mt integrity by producing a long tubular form of mitochondrion in the axon of the glaucomatous glial lamina (Figures 8a and b), whereas glaucomatous D2 mice transduced with AAV2-Null contained fragmented mitochondria in the axons of the glial lamina (Figures 8a and b). Quantitative analysis showed that DRP1 inhibition significantly decreased the number of mitochondria in the axons of the glaucomatous glial lamina (0.40±0.05/mm^2^) compared with glaucomatous D2 mice transduced with AAV2-Null (0.70±0.07/mm^2^; *P*<0.01; Figure 8a). In contrast, DRP1 inhibition significantly increased mt lengths in the axons of the glaucomatous glial lamina (835±31 nm) compared with glaucomatous D2 mice transduced with AAV2-Null (397±11 nm; *P*<0.001; Figure 8a). However, there was no significant difference in mt volume density in the axons between the two groups (Figure 8a), collectively suggesting that DRP1 inhibition preserves axonal integrity by preserving mt integrity. Furthermore, tomographic reconstructions confirmed that there was no difference in the range of cristae shapes or their packing arrangement and density in the axons of the glial lamina between the two groups (Figure 8b and Supplementary Movies S7 and 8), providing evidence of increased mt fission in the axons of the glaucomatous glial lamina with no change to the inner membrane architecture.

## Discussion

There is growing evidence that DRP1-mediated excessive mt fission is associated with neurodegeneration in the CNS.^2, 3, 4, 5, 8, 9, 19, 36^ Moreover, posttranslational modifications of DRP1 are linked to mt fission, synaptic injury or apoptotic cell death in the CNS.^8, 14, 37, 38^ Recent studies demonstrated that DRP1 S616 phosphorylation and mt fragmentation are significantly increased in *S*-nitrosocystein-treated primary cortical neurons *in vitro*,^10^ as well as in amyloid-β-treated primary hippocampal neurons *in vitro* and in brain tissues of Alzheimer's disease.^39^ However, it is unknown whether increasing DRP1 or DRP1 S616 phosphorylation directly induces mt fission-mediated dysfunction in the glaucomatous RGC and degeneration of its axon. In the present study, we observed that increasing total protein expression of DRP1 but not its S616 phosphorylation mediates mt fission in the retina of glaucomatous D2 mice. Indeed, these results are strongly supported by the *in vitro* evidence of the increases of total protein expression of DRP1 but not its S616 phosphorylation in pressurized RGCs, as well as by the induction of mt fission in WT DRP1-overexpressing RGCs. Thus, our present findings suggest that DRP1 S616 phosphorylation-independent DRP1 activity may contribute to mt fission and dysfunction in RGC death during glaucomatous neurodegeneration.

Oxidative stress has been considered an important pathophysiological mechanism in mt dysfunction and glaucomatous neurodegeneration.^22, 23, 25, 40^ We previously demonstrated that elevated IOP triggered mt OPA1 and cytochrome *c* release, and apoptotic cell death in the retinas of glaucomatous D2 mice,^1, 27^ indicating that elevated IOP is associated with the impairment of mt dynamics in the glaucomatous retina. Furthermore, emerging evidence from our group demonstrated that oxidative stress induced by elevated IOP is probably involved in mt dysfunction-mediated RGC death in the retina of glaucomatous D2 mice, as evidenced by increasing SOD2 and Bax, as well as Tfam and oxidative phosphorylation complex IV protein expression.^23^ Together, these results suggest the possibility that oxidative stress could trigger impairments of mt dynamics and function in the glaucomatous retina. Previous studies reported that oxidative stress is associated with DRP1-mediated mt fission and dysfunction in hypertension-induced brain injury^41^ as well as hyperproliferation of vascular smooth muscle cells in pulmonary arterial hypertension.^42^ Of interest, our present findings showed that blocking oxidative stress by CoQ_10_ treatment significantly decreased total DRP1 protein expression but not DRP1 S616 phosphorylation in the retina of glaucomatous D2 mice. Based on our previous findings of CoQ_10_-mediated promotion of RGC survival and preservation of its axon integrity in the ON head (ONH) of glaucomatous D2 mice,^23^ our findings strongly suggest the possibility that oxidative stress induced by elevated IOP may contribute to DRP1-mediated but DRP1 S616 phosphorylation-independent mt fission and dysfunction, and subsequently lead to glaucomatous RGC degeneration.

Inhibition of DRP1 activity by overexpression of DRP1^K38A^ or by selective inhibitors such as mdivi-1 and P110-TAT blocks mt fission and cell death in neurodegeneration.^3, 4, 16, 17, 18, 19, 43^ Furthermore, our previous study demonstrated that mdivi-1 promoted RGC survival in retinal ischemic injury following acute high IOP elevation,^16^ indicating the possibility that DRP1 inhibition may rescue RGCs against mt fission-mediated oxidative stress in pressure-mediated glaucomatous neurodegeneration. In the present study, we found that DRP1 inhibition significantly ameliorated oxidative stress, as evidenced by decreasing SOD2 and Tfam protein expression in the retinas of glaucomatous D2 mice, providing evidence of DRP1-mediated oxidative stress in the glaucomatous retina. As the upregulation of Tfam expression has been proposed as an important mtDNA-related endogenous compensatory mechanism against oxidative stress-mediated retinal neurodegeneration,^23, 35, 44^ our findings suggest that decreasing SOD2 and Tfam protein expression by DRP1 inhibition may reflect mt restoration by blocking DRP1-mediated oxidative stress in the glaucomatous retina. Based on these findings, we suggest that increasing DRP1 has a critical role in oxidative stress-mediated mt dysfunction and DRP1 inhibition may promote RGC survival by blocking mt fission-mediated oxidative stress against glaucomatous neurodegeneration.

In the present study, we found that DRP1 inhibition significantly increased Bad S112 phosphorylation and preserved Bcl-xL protein expression in the retina of glaucomatous D2 mice. Bax is counteracted by Bcl-xL that forms heterodimers with dephosphorylation of Bad, which inactivates Bcl-xL, and Bad phosphorylation eliminates this dimerization, which activates Bcl-xL.^45, 46^ Bcl-xL regulated cell survival by promoting mt adenine-nucleotide exchange and prevented mt hyperpolarization by maintaining mt membrane permability.^47, 48^ Our previous studies have demonstrated that Bcl-xL or Bad S112 phosphorylation was significantly increased in experimental mouse models of retinal ischemia and glaucoma induced by elevated IOP,^23, 35, 49^ suggesting that increasing Bcl-xL or Bad S112 phosphorylation could be an important protective mechanism in the pressure-induced apoptotic pathway of retinal neurodegeneration.^23, 35, 49^ These findings collectively suggest that increasing Bad S112 phosphorylation or preserving Bcl-xL protein expression by DRP1 inhibition may represent important endogenous compensatory mechanisms against mt fission-mediated apoptotic cell death in glaucomatous neurodegeneration. In addition, interestingly, we found that DRP1 inhibition did not change the expression level of Bax protein in the retina of glaucomatous D2 mice. As recent evidence indicates that DRP1 contributes to Bax oligomerization and the cellular apoptotic pathway,^50, 51, 52^ it is possible that DRP1 inhibition may delay or prevent Bax oligomerization without altering Bax protein expression in the glaucomatous retina. Collectively, our findings suggest that DRP1 inhibition protects RGCs by increasing Bad S112 phosphorylation against apoptotic cell death in glaucomatous neurodegeneration.

We previously demonstrated that IOP elevation increased *DNM1* gene expression in the ONH as well as induced mt fission and cristae depletion in the axons of the glial lamina in glaucomatous D2 mice.^1, 53^ Consistently, in the present study, we confirmed mt fission and degraded mitochondria in the degenerative axons of the glial lamina in glaucomatous D2 mice, as evidenced by increased electron-dense lipid deposits and mitophagosome formation. These findings importantly suggest that altered DRP1 activity may contribute to axonal degeneration by impairing the balance of mt dynamics. Of interest, we found for the first time that DRP1 inhibition significantly preserved axonal integrity by restoring the structural integrity of mitochondria in the glial lamina of glaucomatous D2 mice. In good agreement with these findings, there was evidence of DRP1 inhibition-mediated restoration of neurofilament and GFAP immunoreactivity, as well as prevention of hypertrophic astroglial activation in the glial lamina of glaucomatous D2 mice. Furthermore, these results are strongly supported by the present findings of DRP1 inhibition-mediated preservation of axon integrity with intact myelination after the MTZ of the ON in glaucomatous D2 mice. With these results, we suggest the possibility that DRP1 inhibition can promote RGC survival not only by inhibiting mt fission and/or oxidative stress, but also by preserving mt integrity in the axons of the glial lamina in glaucomatous neurodegeneration.

Our findings conclusively demonstrate that not only elevated IOP-induced oxidative stress triggers excessive mt fission, but mt fission also leads to oxidative stress-mediated mt dysfunction in the glaucomatous RGC and degeneration of its axon. Thus, we propose an important vicious cycle involved in glaucomatous neurodegeneration. The cycle starts with elevated IOP producing oxidative stress. This then leads to mt fission that then promotes a specific form of mt dysfunction, which generates further oxidative stress, thus perpetuating the cycle. Further studies addressing altered posttranslational modifications of DRP1-mediated mt dysfunction and oxidative stress in the RGC and its axon may help to elaborate mt dysfunction-mediated pathophysiological mechanisms in glaucomatous neurodegeneration.

## Materials and Methods

### Animals

Adult female C57BL/6, D2 and D2-*Gpnmb*^*+*^ (D2-*Gpnmb*^*+*^) mice (The Jackson Laboratory, Bar Harbor, ME, USA) and untimed pregnant Sprague–Dawley rats (Harlan Laboratories, Indianapolis, IN, USA) were housed in covered cages, fed with a standard rodent diet *ad libitum* and kept on a 12-h light/12-h dark cycle. All procedures concerning animals were in accordance with the Association for Research in Vision and Ophthalmology for the use of animals in research and under protocols approved by the Institutional Animal Care and Use Committee at the University of California, San Deigo.

### Pharmacological treatment

CoQ_10_ was purchased from Kaneka Nutrients (Pasadena, TX, USA). AIN-93G-purified control diet and a diet supplemented with CoQ_10_ were formulated by Harlan Laboratories (Madison, WI, USA).^23, 35^ Four groups of mice were studied: a group of D2-*Gpnmb*^*+*^ mice treated with control diet (*n*=25 mice), a group of D2 mice treated with control diet (*n*=70 mice), a group of D2-*Gpnmb*^*+*^ mice treated with 1% CoQ_10_ diet ((v/v), which equals a daily dose of 1600–2000 mg/kg body weight in 25–30 g mice, *n*=25 mice)^23, 54^ and a group of D2 mice treated with 1% CoQ_10_ diet (*n*=80 mice).^23^

### IOP measurement

IOP elevation onset typically occurs between 5 and 7 months of age, and by 9–10 months of age IOP-linked ON axon loss is well advanced.^1, 55, 56^ IOP measurement was performed as previously described.^33, 57^ Each of the 9-month-old D2 mice used in this study had a single IOP measurement (to confirm the development of spontaneous IOP elevation exceeding 20 mm Hg; *n*=25 for D2 mice, *n*=33 for D2 mice transduced with AAV2-GFP or AAV2-Null, *n*=59 for D2 mice transduced with AAV2-DRP1^K38A^). In addition, each of the non-glaucomatous control C57BL/6 or D2-*Gpnmb*^*+*^ mice (*n*=21) used in this study had a single IOP measurement. After anesthesia with intraperitoneal (IP) injection of a mixture of ketamine (100 mg/kg, Ketaset, Fort Dodge Animal Health, Fort Dodge, IA, USA) and xylazine (9 mg/kg, TranquiVed, Vedeco, Inc., St. Joseph, MO, USA), a sterilized, water-filled microneedle with an external diameter of 50–70 *μ*m was used to cannulate the anterior chamber. The microneedle was then repositioned to minimize corneal deformation and to ensure that the eye remained in its normal position. The microneedle was connected to a pressure transducer (Blood Pressure Transducer, WPI, Sarasota, FL, USA), which relayed it signal to a bridge amplifier (Quad Bridge, AD Instruments (ADI), Castle Hill, NSW, Australia). The amplifier was connected to an analog-to-digital converter (Power Laboratory, ADI) and a computer (G4 Macintosh, Apple Computer, Inc., Cupertino, CA, USA). After measuring the IOP, we used the glaucomatous D2 mice that had spontaneous IOP elevation exceeding 20 mm Hg^1, 57^ and the mice were confirmed by examining the ON axon damage.^34, 55, 56, 58^

### Tissue preparation

Mice were anesthetized with IP injection of a mixture of ketamine (100 mg/kg, Ketaset, Fort Dodge Animal Health) and xylazine (9 mg/kg, TranquiVed, VEDCO Inc.) before cervical dislocation. For immunohistochemistry, the retinas and ONHs were dissected from the choroids and fixed with 4% paraformaldehyde in phosphate buffered saline (PBS, pH 7.4) for 2 h at 4 °C. After several washes in PBS, the retinas were dehydrated through graded ethanols and embedded in polyester wax. For western blot analyses, extracted retinas were immediately used.

### Purification of RGCs and pressure system *in vitro*

RGCs from postnatal day 5 of Sprague–Dawley rat were purified by immunopanning and were cultured in serum-free defined growth medium as previously described.^59^ A pressurized incubator was used to expose the cells to elevated hydrostatic pressure as previously described.^60, 61^ The plexiglass pressure chamber was connected via a low-pressure two-stage regulator (Gilmont Instruments, Barnant Company, Barrington, IL, USA) to a certified source of 5% CO_2_/95% air (Airgas, Inc., San Diego, CA, USA). Measurements for pH, pCO_2_ and pO_2_ analysis were done using a portable blood gas analyzer (iSTAT Corp., East Windsor, NJ, USA) as previously described.^60^

### Transfection of plasmid constructs

The pDsRed2-Mito was obtained from Clontech (Mountain View, CA, USA) and the pcDNA3-DRP1^K38A^ plasmid in baculovirus expression vector (US National Center for Biotechnology Information accession number NM_005690.3) was provided by Dr AM van der Bliek. For transfection of primary RGCs, 100 *μ*l of Nucleofector Solution (Lonza, Allendale, NJ, USA) was mixed with 1 × 10^6^ cells and then 1 *μ*g of pcDNA3 and pcDNA3-DRP1 were transfected using a Nucleofector II/2b Device (Lonza).

### Transduction of recombinant AAV2 constructs

The AAV2-cytomegalovirus (CMV)-pDsRed2-Mito (2.9 × 10^11^ GC/ml) and AAV2-CMV-DRP1^K38A^ (5 × 10^11^ GC/ml) were produced using the pAAV-CMV-shuttle by Applied Viromics (Fremont, CA, USA). The AAV2-Null and AAV2-CMV-GFP (1 × 10^12^ GC/ml) were purchased from Applied Viromics. The 7-month-old pre-glaucomatous D2 and D2-*Gpnmb*^*+*^ mice were anesthetized with IP injection of a mixture of ketamine/xylazine and topical 1% proparacaine eye drops. A glass needle was used to inject a total of 2 *μ*l AAV2 constructs into the vitreous humor. Two injections of AAV2 constructs, each 1 *μ*l, were delivered in one eye. Injections were given slowly over 1 min and the needle was maintained in position for an additional 10 min to minimize vector loss through the injection tract. At age of 9 months, the mice were euthanized by an IP injection of a mixture of ketamine/xylazine and the retina and ONH tissues were prepared as above.

### Western blot analysis

Retinas were dissected from the sclera of mice. The retinal tissues or primary RGCs were then immediately homogenized in a glass-teflon Potter homogenizer in lysis buffer (20 mM Hepes, pH 7.5, 10 mM KCl, 1.5 mM MgCl_2_, 1 mM EDTA, 1 mM DTT, 0.5% CHAPS/complete protease inhibitors; Roche Biochemicals, Indianapolis, IN, USA). Ten micrograms of pooled retinal protein (*n*=4 retinas/group) from each group were separated by PAGE and electrotransferred to polyvinylidenedifluoride membranes. The membrane was blocked with 5% non-fat dry milk/0.5% Tween-20/PBS for 1 h and subsequently incubated with the primary antibodies overnight. The primary antibodies included mouse monoclonal anti-DRP1 antibody (1 : 1000, BD Transduction Laboratories, San Diego, CA, USA), rabbit polyclonal anti-phospho-DRP1 S616 antibody (1 : 1000, Cell Signaling, Danvers, MA, USA), rabbit polyclonal anti-SOD2 antibody (1 : 3000; Santa Cruz Biotechnology, Santa Cruz, CA, USA), rabbit polyclonal anti-Tfam antibody (1 : 3000, Santa Cruz Biotechnology), rabbit polyclonal anti-Bax antibody (1 : 500, Santa Cruz Biotechnology), mouse monoclonal anti-phospho-Bad S112 antibody (1 : 500, Cell Signaling) and mouse monoclonal anti-actin antibody (1 : 5000; Millipore, Billerica, MA, USA). After several washes in Tween/PBS, the membranes were incubated with peroxidase-conjugated goat anti-mouse IgG (1 : 2000; Bio-Rad, Hercules, CA, USA) or goat anti-rabbit IgG (1 : 2000; Bio-Rad) and developed using chemiluminescence detection (ECL Plus; GE Healthcare Bio-Science, Piscataway, NJ, USA). The scanned film images were analysed by ImageJ (http://rsb.info.nih.gov/ij/) and band densities were normalized to the band densities for actin.

### Immunohistochemistry and immunocytochemistry

Immunohistochemical staining of 7 *μ*m wax sections of full thickness retina and ONH or immunocytochemical staining of cultured RGCs were performed. Sections from wax blocks from each group (*n*=4 retinas or ONHs/group) and coverslips from the cultures (*n*=3 per group) were used for immunohistochemical or immunocytochemical analysis, respectively. Primary antibodies included goat polyclonal anti-Brn3a antibody (1 : 500, Santa Cruz Biotechnology), mouse monoclonal anti-DRP1 antibody (1 : 50, Santa Cruz Biotechnology), mouse monoclonal anti-phospho-neurofilament H (SMI-32) antibody (1 : 500; Covance, Inc., San Diego, CA), rabbit polyclonal anti-SOD2 antibody (1 : 300, Santa Cruz Biotechnology), rabbit polyclonal anti-Tfam antibody (1 : 300, Santa Cruz Biotechnology), guinea pig polyclonal anti-GFAP antibody (1 : 500, Advanced ImmunoChemical, Long Beach, CA, USA) or mouse monoclonal anti-neurofilament (1 : 100, Sigma, St. Louis, MO, USA). To prevent nonspecific background, tissues were incubated in 1% bovine serum albumin/PBS for 1 h at room temperature before incubation with the primary antibodies for 16 h at 4 °C. After several wash steps, the tissues were incubated with the secondary antibodies, Alexa Fluor 488 dye-conjugated goat anti-mouse IgG (1 : 100, Invitrogen, Carlsbad, CA, USA), Alexa Fluor 488 dye-conjugated goat anti-rabbit IgG (1 : 100, Invitrogen), Alexa Fluor 568 dye-conjugated donkey anti-goat IgG (1 : 100, Invitrogen) or Cy5-conjugated anti-guinea pig IgG antibody (1 : 100, Jackson ImmunoResearch Laboratories, West Grove, PA, USA) for 4 h at 4 °C and subsequently washed with PBS. The sections were counterstained with the nucleic acid stain Hoechst 33342 (1 *μ*g/ml; Invitrogen) in PBS. Images were acquired with confocal microscopy (Olympus FluoView1000, Olympus, Tokyo, Japan).

### Whole-mount immunohistochemistry

Retinas from enucleated eyes were dissected as flattened whole mounts from 9-month-old glaucomatous D2 mice transduced with AAV2-GFP or AAV2-DRP1^K38A^. Retinas were immersed in PBS containing 30% sucrose for 24 h at 4 °C. The retinas were blocked in PBS containing 3% donkey serum, 1% bovine serum albumin, 1% fish gelatin and 0.1% Triton X-100, and incubated with goat polyclonal anti-Brn3a antibody (1 : 500, Santa Cruz Biotechnology) and/or mouse monoclonal anti-phospho-neurofilament H (SMI-32) antibody (1 : 500; Covance, Inc.) for 3 days at 4 °C. After several wash steps, the retinas were incubated with the secondary antibodies, Alexa Fluor 488 dye-conjugated goat anti-rabbit IgG (1 : 100, Invitrogen) and/or Alexa Fluor-568 donkey anti-goat IgG antibody (1 : 100, Invitrogen) for 24 h, and subsequently washed with PBS. Images were captured with a spinning-disc confocal microscope (Olympus America, Inc., Center Valley, PA, USA).

### Quantitative analysis for RGC counting

To count RGCs labeled with Brn3a, each retinal quadrant was divided into three zones by central, middle and peripheral retina (one sixth, three sixths and five sixths of the retinal radius). RGC densities were measured in 24 distinct areas (2 areas at central, middle and peripheral per retinal quadrant) per condition by two investigators in a masked manner and the scores were averaged. To count RGCs labeled with AAV2-GFP and Brn3a, each retinal quadrant was divided into three zones by central, middle and peripheral retina (one sixth, three sixths and five sixths of the retinal radius). RGC densities were measured in four distinct areas in the middle retina (three sixths of the retinal radius) and the scores were averaged.

### Cell viability and ROS measurements

Cell viability was measured in primary RGCs cultured in 96-well plates using MTT (3-[4,5-dimethylthiazol-2yl]-2,5-diphenyl tetrazolium bromide) according to the manufacturer's recommendations (Cell Proliferation Kit 1, Roche Diagnostics, Indianapolis, IN, USA). Briefly, cells were grown in 96-well plates with a final volume of 100 *μ*l culture medium per well. At 3 days after exposure with elevated hydrostatic pressure, 10 *μ*l of the MTT labeling reagent (final concentration 0.5 mg/ml) was added to each well and the cultures were incubated in a conventional CO_2_ incubator at 37 °C for 4 h. Next, 100 *μ*l of the solubilization solution was added into each well and the plates were incubated for 16 h in a humidified atmosphere of 5% CO_2_ incubator at 37 °C. Absorbance at 560 nm was then measured using a microplate reader (Spectra MAX, Molecular Devices, Corp., Sunnyvale, CA, USA). Data are presented as the percentage of cell viability in same day control wells, respectively.^60^ The intracellular ROS was measured by 5-(and-6)-chloromethyl-2′,7′-dichlorodihydrofluorescein diacetate, acetyl ester (CM-H_2_DCFDA, Life Technologies, Grand Island, NY, USA), a chloromethyl derivative of H_2_DCFDA, useful as an indicator for ROS in cells. Briefly, ONH astrocytes were plated on a six-well plate (1.7 × 10^4^/well), and after 24 h cells were pre-incubated with 50 *μ*g/ml CoQ_10_ for 24 h and then exposed to H_2_O_2_. The cells were detached with Trypsin/EDTA and loaded with 20 *μ*M CM-H_2_DCFDA at 37 °C for 20 min, and then fluorescence of the sample was measured immediately using flow cytometry (BD FACSCanto II, BD Bioscience, San Diego, CA, USA). Each set of data was collected from multiple replicate dishes of each experimental group (*n*=3).

### Transmission electron microscopy

For conventional EM, two eyes from each group (*n*=2 mice) were fixed via cardiac perfusion with 2% paraformaldehyde, 2.5% glutaraldehyde (Ted Pella, Redding, CA, USA) in 0.15 M sodium cacodylate (pH 7.4, Sigma) solution at 37 °C and placed in pre-cooled fixative of the same composition on ice for 1 h. The following procedure was used to optimize mt structural preservation and membrane contrast.^62^ The retinas were dissected in a solution of 0.15 M sodium cacodylate plus 3 mM calcium chloride (pH 7.4) on ice and then postfixed with a 1% osmium tetroxide, 0.8% potassium ferrocyanide, 3 mM calcium chloride in 0.1 M sodium cacodylate solution (pH 7.4) for 1 h, washed with ice-cold distilled water, poststained with 2% uranyl acetate solution at 4 °C, dehydrated using graded ethanols and embedded in Durcupan resin (Fluka, St. Louis, MO, USA). A similar procedure was used for cultured primary RGCs from each group. Ultrathin (70 nm) sections were poststained with uranyl acetate and lead salt solutions, and evaluated using a JEOL 1200FX transmission EM (Japanese Electron Optics, Ltd., Tokyo, Japan) operated at 80 kV. Images were recorded on film at × 8000 magnification. The negatives were digitized at 1800 dpi using a Nikon Cool scan system, giving an image size of 4033 × 6010 pixel array and a pixel resolution of 1.77 nm.^62^ For quantitative analysis, the number of mitochondria was normalized to the total area occupied by axons in each image, which was measured using ImageJ (http://rsb.info.nih.gov/ij/). Mitochondrial lengths were measured with ImageJ. The mt volume density, defined as the volume occupied by mitochondria divided by the volume occupied by the axoplasm, was estimated using stereology as follows. A 112 × 112 square grid (112 × 112 chosen for ease of use with Photoshop) was overlaid on each image loaded in Photoshop (Adobe Systems, Inc., San Jose, CA, USA) and the mitochondria and axoplasm lying under intercepts were counted. The relative volume of the mitochondria was expressed as the ratio of intercepts coinciding with this organelle relative to the intercepts coinciding with axoplasm.

### Electron microscope tomography

Sections of ONH tissues or cultured RGCs from each group were cut at thicknesses of 400–500 nm. For each reconstruction, a series of images at regular tilt increments was collected with a JEOL 4000EX intermediate-voltage electron microscope (Japanese Electron Optics, Ltd.) operated at 400 kV. The magnification was × 12 000 and the pixel resolution was 1.2 nm. The IMOD package was used for a rough alignment with the fine alignment and reconstruction performed using the TxBR package. Volume segmentation was performed by manual tracing in the planes of highest resolution with the program Xvoxtrace.^63^ The mt reconstructions were visualized using Analyze (Mayo Foundation, Rochester, MN, USA) or the surface-rendering graphics of Synu (National Center for Microscopy and Imaging Research, San Diego, CA, USA) as previously described.^63^ These programs allow one to step through slices of the reconstruction in any orientation and to track or model features of interest in three dimensions. Measurements of mt outer, inner boundary and cristae membrane surface areas and volumes were made within segmented volumes by the programs Synuarea and Synuvolume, respectively (National Center for Microscopy and Imaging Research). These were used to determine the cristae density, defined as the ratio: sum of the cristae volumes divided by the mt volume. Movies of the tomographic volume were made using Amira (FEI, Hillsboro, OR, USA).

### Serial block-face scanning electron microscopy

The ONHs were washed in cacodylate buffer for 2 h at 4 °C and placed in cacodylate buffer containing 2% OsO_4_/1.5% potassium ferrocyanide for 3 h at room temperature. Following several washing steps, the ONHs were dehydrated in a series of ice-cold ethanol solutions followed by ice-cold dry acetone for 10 min. The ONHs were placed in acetone at room temperature for 10 min and then infiltrated with an ascending series of Durcupan:acetone solutions. The ONHs were infiltrated with 100% Durcupan and then cured at 60 °C for 2 days. The ONHs were trimmed to remove excess plastic and attached to an aluminum pin, grounded with silver paint and sputter coated with gold–palladium before imaging. Specimens were imaged on a FEI Quanta FEG equipped with a 3View serial block-face system (Gatan, Inc., Pleasanton, CA, USA). Specimens were imaged at high vacuum with 2.5-kV beam current and 70-nm sectioning thickness. A 2D montage was collected at each Z plane to increase field of view. Once a volume was collected, the histograms for slices throughout the volume stack were normalized to correct for the drift in image intensity during acquisition. Digital micrograph files (.dm3) were converted to MRC format. The stacks were converted to eight bit and volumes were manually traced for reconstruction and analysis. To analyze these volumes, we used the publicly available software package IMOD, specifically developed for the visualization and analysis of EM data sets in three dimensions (http://bio3d.colorado.edu/imod/)^64^ and stereology was performed using a custom plug-in for IMOD.^65^

### Statistical analysis

Data were presented as the mean±S.D. or mean±S.E.M. Comparison of two or three experimental conditions was evaluated using the unpaired Student's *t*-test or one-way analysis of variance and the Bonferroni *t*-test. *P*<0.05 was considered to be statistically significant.

## Abbreviations

AAV2: adeno-associated virus serotype 2
CNS: central nervous system
CoQ_10_: coenzyme Q10
D2: DBA/2J
DRP1: dynamin-related protein 1
DRP1^K38A^: dominant-negative DRP1 K38A mutant
GCL: ganglion cell layer
GFAP: glial fibrillary acidic protein
GFP: green fluorescent protein
IOP: intraocular pressure
IP: intraperitoneal
MIP: maximum intensity projection
mt: mitochondrial
MTZ: myelination transition zone
ON: optic nerve
ONH: ON head
OPL: outer plexiform layer
POAG: primary open-angle glaucoma
RGC: retinal ganglion cell
ROS: reactive oxygen species
S112: serine 112
S616: serine 616
SBEM: serial block-face scanning electron microscopy
SOD2: superoxide dismutase 2
Tfam: mt transcription factor A
WT: wild type
3D: three-dimensional

## Supplementary Material

Supplemementary Movie S1

Supplemementary Movie S2

Supplemementary Movie S3

Supplemementary Movie S4

Supplemementary Movie S5

Supplemementary Movie S6

Supplemementary Movie S7

Supplemementary Movie S8

Supplemementary Information
