# Supplementary material for: DRP1 inhibition rescues retinal ganglion cells and their axons by preserving mitochondrial integrity in a mouse model of glaucoma
Source: Cell Death Dis. 2015 Aug 6;6(8):e1839–. doi: 10.1038/cddis.2015.180 (PMC4558491; doi:10.1038/cddis.2015.180)
Supplement: Supplemementary Information [file cddis2015180x9.pdf]

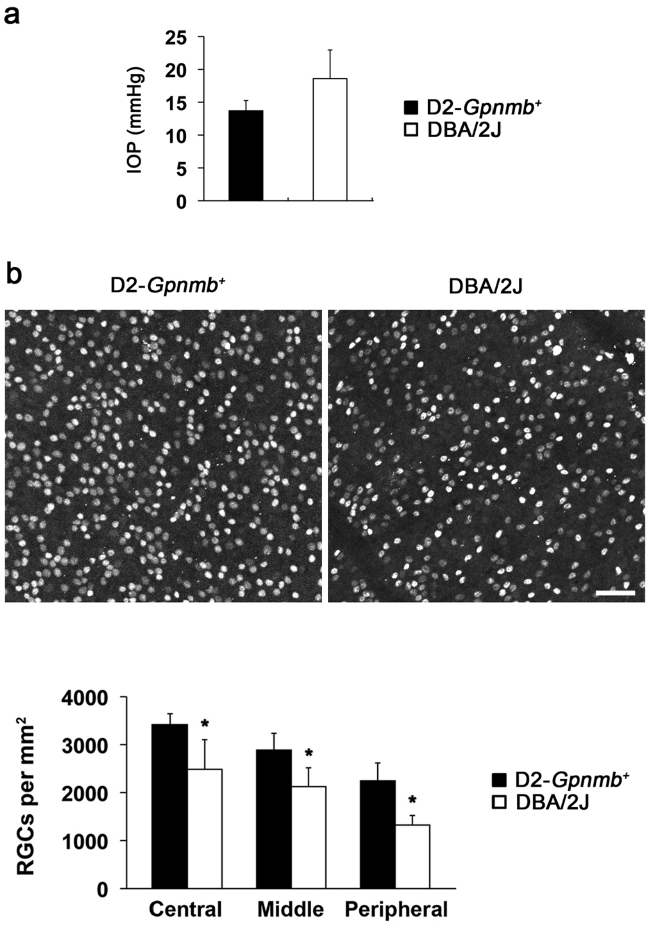

Supplementary Figure S1. RGC loss in the retina of glaucomatous DBA/2J mice. (a) The representative graph of average IOPs in D2-*Gpnmb*$^{+}$ and glaucomatous DBA/2J mice (n = 25 mice/group). Values are mean ± SD. (b) The representative images from the middle area of retinal flatmounts in D2-*Gpnmb*$^{+}$ and glaucomatous DBA/2J mice. Quantitative analysis showed a significant loss of RGC in glaucomatous DBA/2J mice. Mean RGC density per retina for each group is presented. Values are mean ± SD. *Significant at $P < 0.05$ compared with D2-*Gpnmb*$^{+}$ mice. Scale bar: 50 µm (all panels).

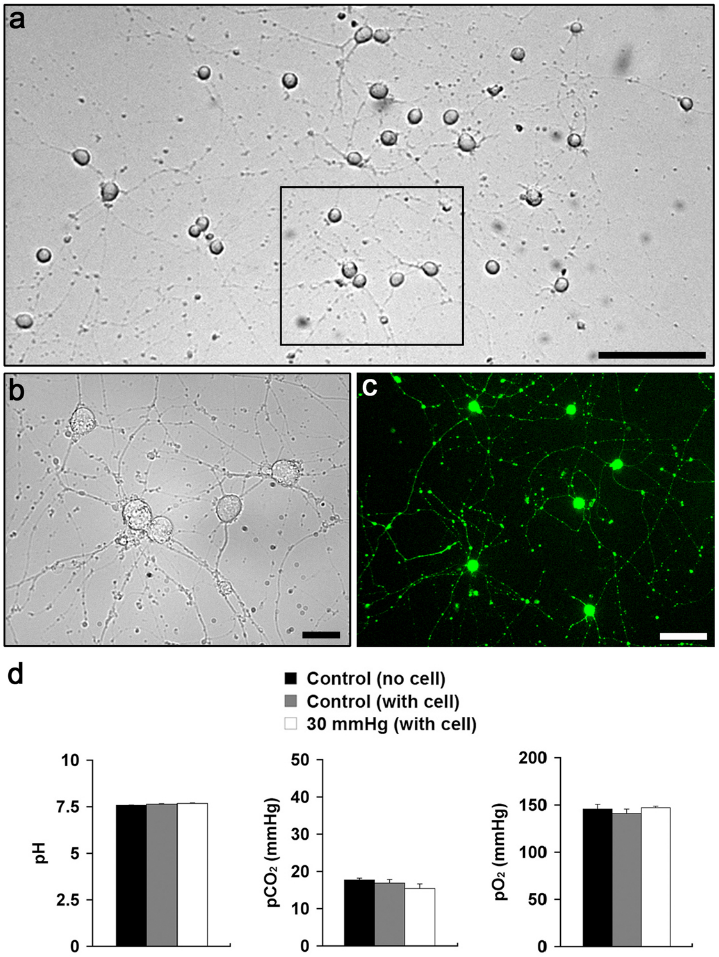
a
b
c
d
Control (no cell)
Control (with cell)
30 mmHg (with cell)
pH
10
7.5
5
2.5
0
pCO2 (mmHg)
50
40
30
20
10
0
pO2 (mmHg)
200
150
100
50
0

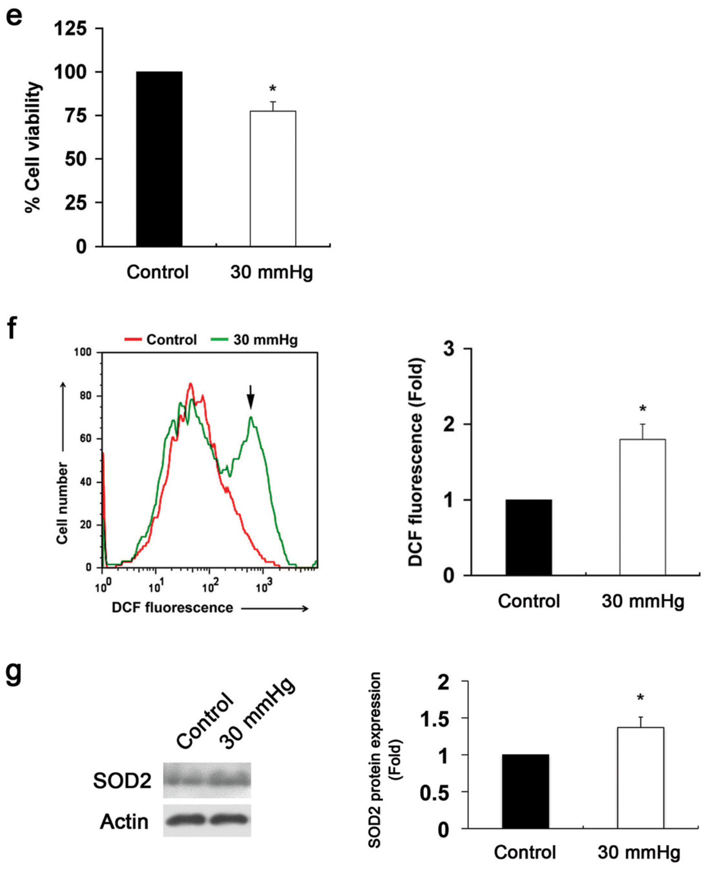

Supplementary Figure S2. Elevated hydrostatic pressure induces oxidative stress in cultured RGCs *in vitro*. Gas analysis of RGC culture media. (a) Cultured RGCs at 3 days without elevated hydrostatic pressure. Scale bar: 100 µm. (b) High magnification showed healthy cell bodies and various dendritic trees (solid box in a). Scale bar: 10 µm. (c) Cell viability was confirmed by calcein AM staining without elevated hydrostatic pressure. Scale bar: 50 µm. (d) Measurement of pH, pCO_2, and pO_2 after 3 days with or without elevated hydrostatic pressure application. Values are mean ± SD. (e) Cell viability was significantly decreased in pressurized RGCs compared with non-pressurized control RGCs ($n = 8$ replicate wells/group). (f) Overlaying histograms of DCF fluorescence in non-pressurized and pressurized RGCs. Arrow indicates DCF fluorescence shift following elevated pressure. Note that pressurized RGCs showed a significant increase of ROS generation ($n = 3$ replicate dishes/group). (g) SOD2 protein expression was significantly increased in pressureized RGCs compared with non-pressurized control RGCs. Values are mean ± SD. *Significant at $P < 0.05$ compared with non-pressurized control RGCs.

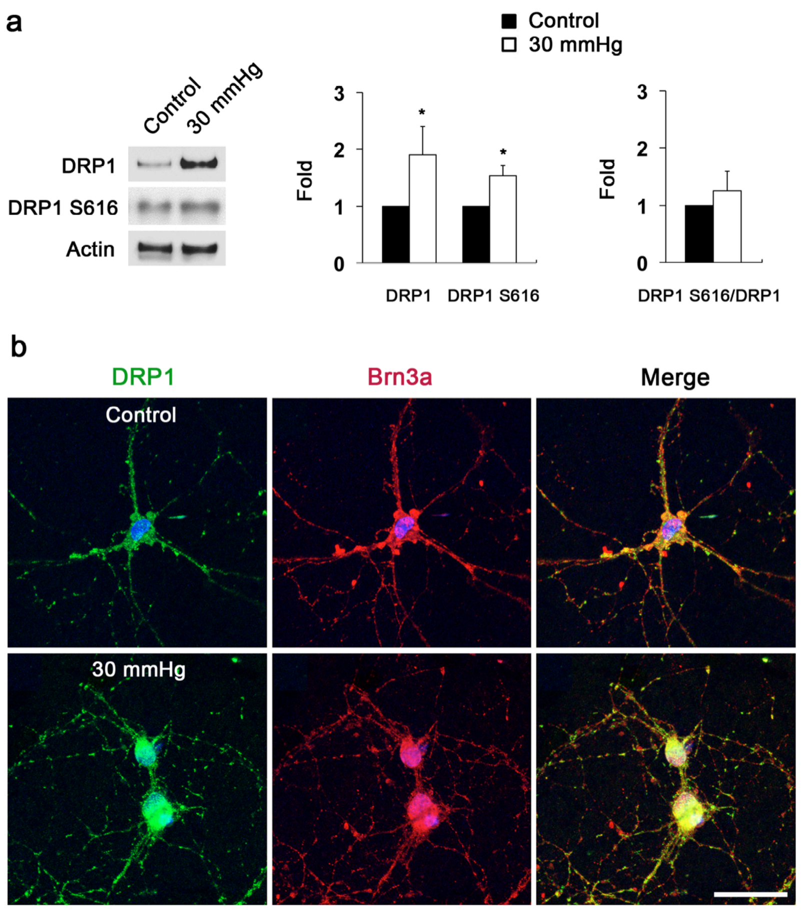

Supplementary Figure S3. Elevated hydrostatic pressure upregulates DRP1 protein expression but not DRP1 S616 phosphorylation in cultured RGCs *in vitro*. (a) DRP1 protein expression but not DRP1 S616 phosphorylation were significantly increased in pressurized RGCs compared with non-pressurized control RGCs. Values are mean ± SD. *Significant at $P < 0.05$ compared with non-pressurized control RGCs. (b) DRP1 and Brn3a double immunocytochemistry. The representative images showed that DRP1 immunoreactivity was increased in the somas of Brn3a-positive pressurized RGCs compared with non-pressurized control RGCs. Scale bar: 20 µm (all panels).

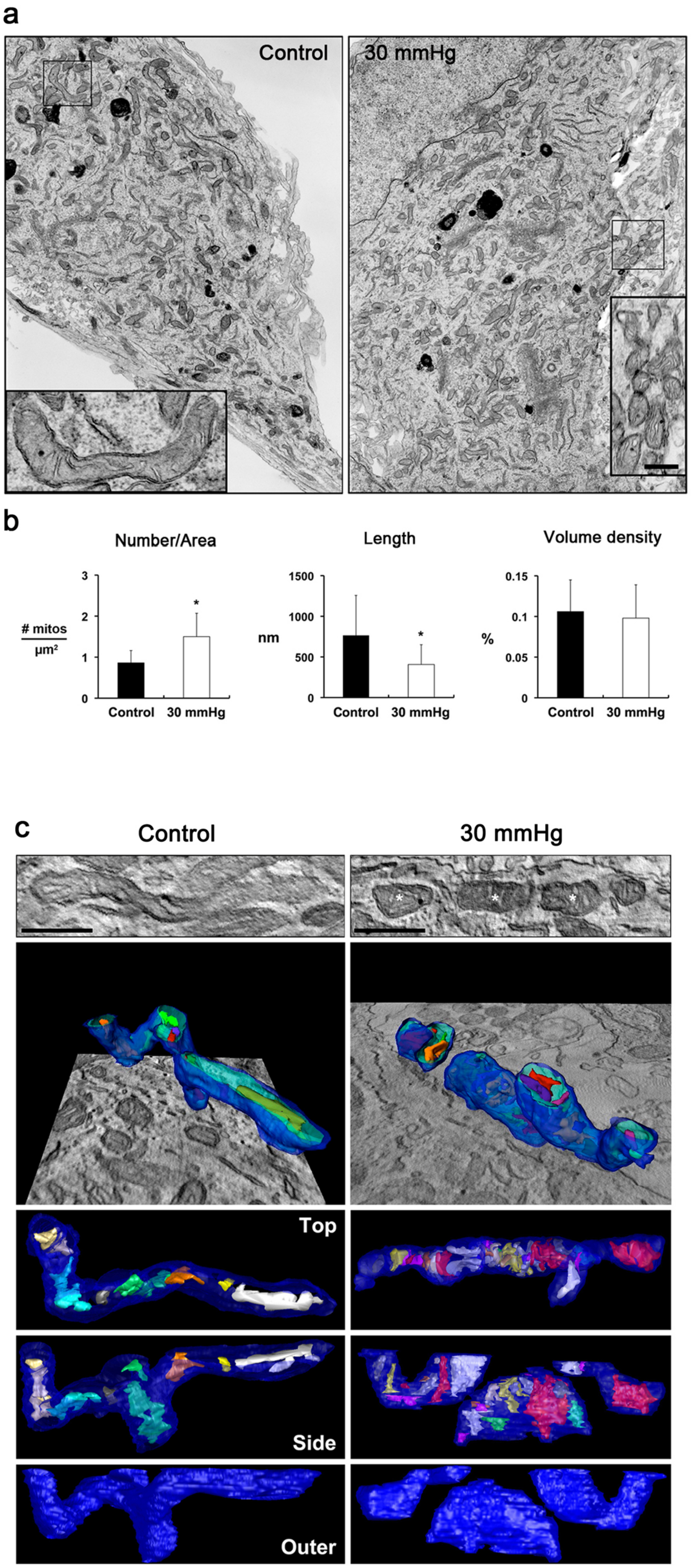
a
Control
30 mmHg
b
Number/Area
Length
Volume density
mitos
μm²
nm
%
3
2
1
0
1500
1000
500
0
0.15
0.1
0.05
0
*
*
Control
30 mmHg
c
Control
30 mmHg
Top
Side
Outer

Supplementary Figure S4. Elevated hydrostatic pressure triggers mitochondrial fission in cultured RGCs *in vitro*. (a) The representative 2D images from TEM analysis showed that the soma in non-pressurized control RGCs had elongated mitochondrion. In contrast, the soma in pressurized RGC had small rounded mitochondria. Scale bar: 500 nm (all panels). (b) Quantitative analysis showed that the number of mitochondria was significantly increased in pressurized RGCs compared with non-pressurized control RGCs. In contrast, mitochondrial lengths were significantly decreased in pressurized RGCs compared with non-pressurized control RGCs. However, there was no difference in mitochondrial volume density between non-pressurized control and pressurized RGCs. Values are mean ± SD. *Significant at $P < 0.05$ compared with non-pressurized control RGCs. (c) 3D reconstruction of mitochondrial fission and cristae. 1.36-nm thick slices through the middle of EM tomographic volumes of mitochondria are shown. Surface-rendered volumes of the segmented mitochondria provide information concerning shape and cristae architecture. The outer mitochondrial membrane is shown in blue (made translucent to better visualize the cristae) and cristae are in various colors. Scale bar: 500 nm (all panels).

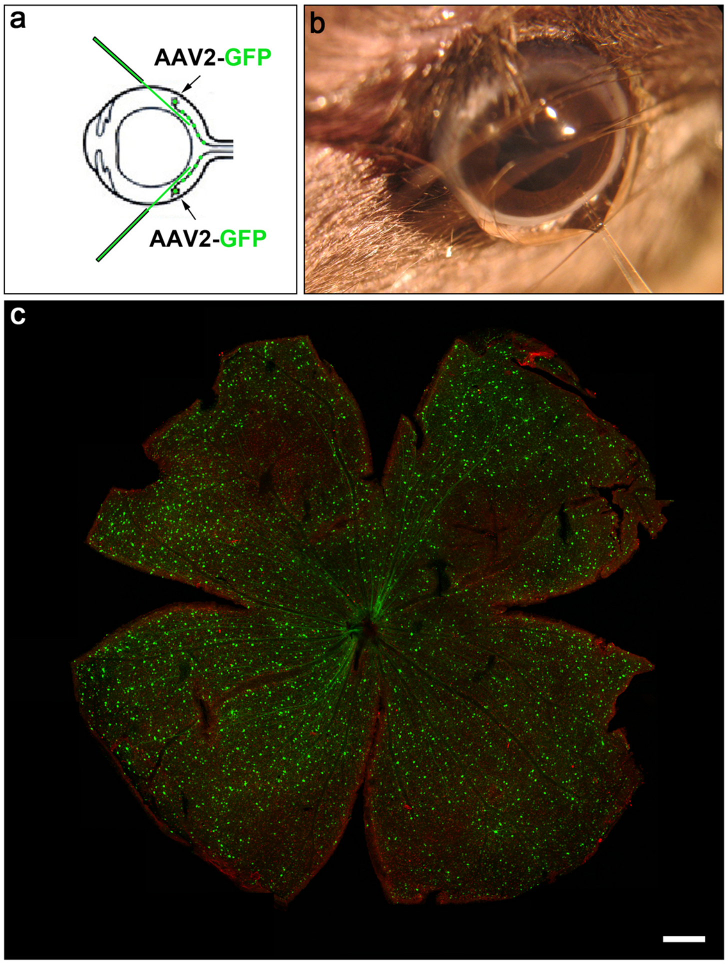
a
AAV2-GFP
AAV2-GFP
b
c

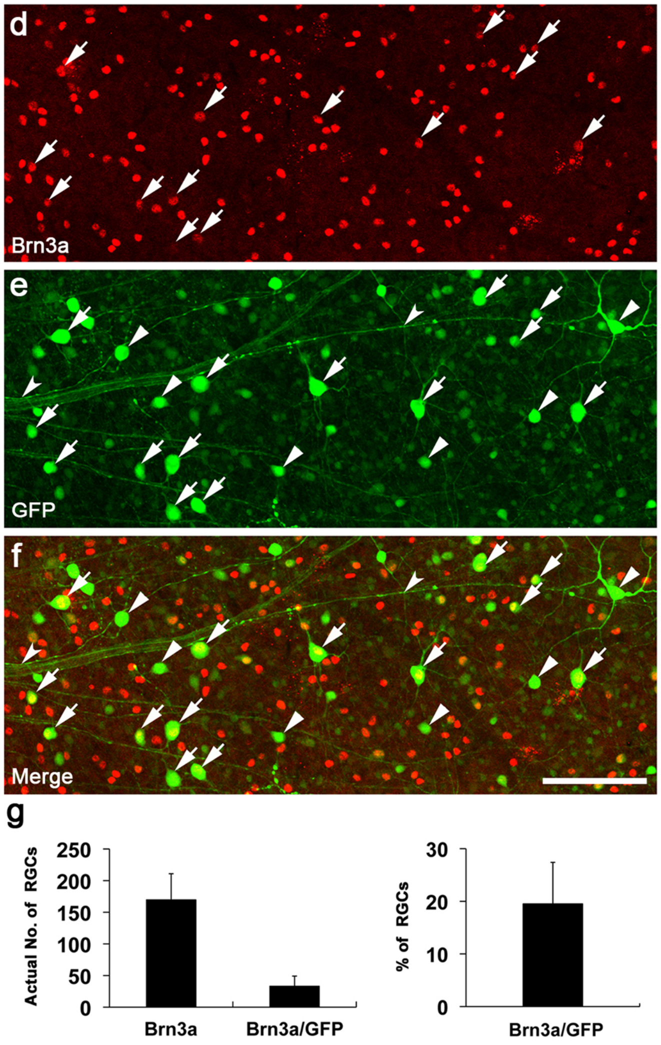

Supplementary Figure S5. GFP expression in the whole retina in glaucomatous DBA/2J mice. (a) Diagram for double-intravitreal injections using AAV2-GFP. (b) Actual photograph for intravitreal injection using a glass needle. (c) Note that GFP expression was observed throughout the whole retina. Scale bar, 100 µm. (d-f) Double labeling of Brn3a and GFP using whole-mount immunohistochemistry. Brn3a (red, d), GFP (green, e) and Merged image (f). Note that GFP expression was observed in the somas (arrows) and axons (concave arrowheads) of RGCs, as well as the entire dendritic tree and cell bodies of displaced amacrine cells (arrowheads) in the GCL. (g) Quantitative analysis of tansduction efficiency of AAV2-GFP. Mean number of RGCs labeled with both GFP and Brn3a was 19.5 ± 7.9%. Values are mean ± SD (n = 5 retinal flatmounts). The actual number of RGCs was presented by 250 pixels^2/300dpi. Scale bar: 100 µm (all panels).

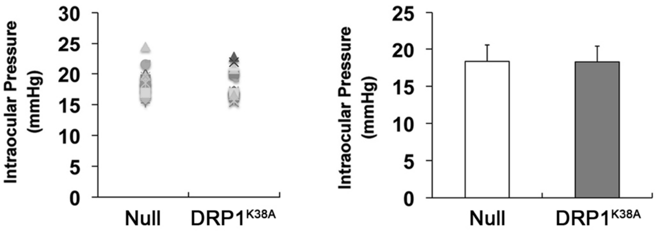

Supplementary Figure S6. IOP measurement. The representative graph for actual IOPs (left) and the representative graph for average IOP (right) (*n* = 25 mice/group). Values are mean ± SD.

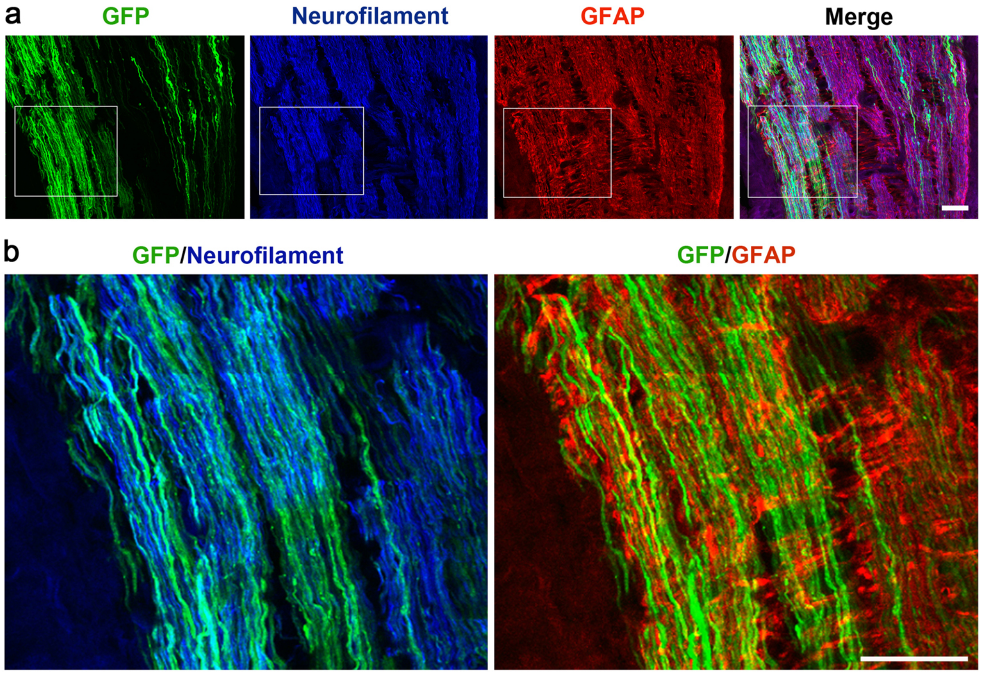

Supplementary Figure S7. Transduction of AAV2-GFP in the axons of the glial lamina of glaucomatous DBA/2J mice. (a and b) Triple labeling with GFP (green), neurofilament (blue) and GFAP (red). Expression of GFP in the axons of the ONH axons in 6-mo-old DBA/2J mice transduced with AAV2-GFP (a). Note that GFP expression was observed in neurofilament-positive ONH axons (cyan) but not in GFAP-positive ONH astrocytes (red, B). Scale bar: 100 µm (all panels).

Supplemenaty Table S1. RGC loss in the central, middle and peripheral retina from 9-mo-old glaucomatous DBA/2J mice.

Strain	Age (Months)	Mean IOP (mmHg)	RGC density per retina ($RGCs/mm^2$)		
			Central	Middle	Peripheral
D2-*Gpnmb*$^+$	9	13.5 ± 1.6	3419 ± 226	2889 ± 347	2247 ± 372
DBA/2J	9	18.6 ± 4.4	2487 ± 616*	2125 ± 393*	1324 ± 200*

Values are given as means ± SD. Comparison of two experimental conditions was evaluated using the unpaired Student's *t*-test. *Significant at $P < 0.05$ compared with 9-mo-old D2-*Gpnmb*$^+$ mice.

Supplementary Table S2. Measurement of pH, pCO_2, pO_2 following 3 days with or without pressure (30 mmHg) application in primary RGC cultures.

Treatment	Control (No cell)	Control (with cell)	Pressure (with cell)
Time (days)	3	3	3
pH	7.58 ± 0.01	7.64 ± 0.03	7.68 ± 0.03
pCO_2 (mmHg)	17.7 ± 0.5	16.9 ± 0.95	15.4 ± 1.24
pO_2 (mmHg)	145.6 ± 5.13	141 ± 4.58	147 ± 1.73

Values are given as means ± SD. Comparison of three experimental conditions was evaluated using the one-way analysis of variance and the Bonferroni *t*-test. Statistical analysis showed no significant difference in pH, pCO_2 or pO_2 between the pressure and control RGC culture media ($P > 0.5$, n = 3).

Supplementary Table S3. RGC survival in the central, middle and peripheral retina from 9-mo glaucomatous D2 mice transduced with AAV2-DRP1^{K38A}.

Strain	Age (Months)	Treatment (AAV2)	Animal (n)	RGC density per retina ($RGCs/mm^2$)		
				Central	Middle	Peripheral
DBA/2J	9	GFP	9	2456 ± 532	2094 ± 608	1317 ± 508
DBA/2J	9	DRP1^{K38A}	11	3094 ± 454*	2702 ± 485*	1833 ± 562*

Values are given as means ± SD. Comparison of two experimental conditions was evaluated using the unpaired Student *t*-test. *Significant at $P < 0.05$ compared with 9-mo-old glaucomatous DBA/2J mice transduced with AAV2-GFP

Supplementary Movies Legends

Supplementary Movie S1: The MIP of SBEM volume from the glial lamina of control C57BL mouse.

Supplementary Movie S2: The MIP of SBEM volume from the glial lamina of glaucomatous DBA/2J mouse.

Supplementary Movie S3: 3D reconstruction of elongated mitochondria in the axon from SBEM volume in the glial lamina of control C57BL mouse.

Supplementary Movie S4: 3D reconstruction of mitochondrial fission in the axon from SBEM volume in the glial lamina of glaucomatous DBA/2J mouse.

Supplementary Movie S5: 3D reconstruction of an elongated mitochondrion and its cristae in non-pressurized control RGC *in vitro*.

Supplementary Movie S6: 3D reconstruction of mitochondrial fission and their cristae in pressurized RGC *in vitro*.

Supplementary Movie S7: 3D reconstruction of mitochondrial fission and their cristae in the axon of the glial lamina in glaucomatous DBA/2J mice transduced with AAV2-Null.

Supplementary Movie S8: 3D reconstruction of an elongated mitochondrion and its cristae in the axon of the glial lamina in glaucomatous DBA/2J mice transduced with AAV2-DRP1^{K38A}.
